# Advances and directions in chemotherapy using implantable port systems for colorectal cancer: a historical review

**DOI:** 10.1007/s00595-013-0672-8

**Published:** 2013-07-28

**Authors:** Yasuhiro Inoue, Masato Kusunoki

**Affiliations:** Department of Gastrointestinal and Pediatric Surgery, Division of Reparative Medicine, Institute of Life Sciences, Mie University Graduate School of Medicine, 2-174 Edobashi, Tsu, Mie 514-8507 Japan

**Keywords:** Colorectal cancer, Chemotherapy, Implantable port system

## Abstract

With the recent advances in chemotherapy for colorectal cancer, the prognosis for patients with metastatic colorectal cancer has been significantly improved. The development of the implantable port system has also enabled patients to receive multiagent chemotherapy with a more satisfactory quality of life. Historically, chemotherapy using implantable port systems was begun to obtain an oncological benefit in the treatment of locoregional cancer. In the 1950s, there was an increasing interest in perfusion techniques for the application of chemotherapeutic agents, such as nitrogen mustard, in the locoregional treatment of metastatic cancer. Among them, the treatment of liver metastasis has interested oncologists for many years. On the other hand, implantable devices were developed during the intervening decades that have enabled patients with colorectal cancer with liver metastases to be treated effectively using hepatic arterial infusion; which became more common in the 1980s. The treatment of metastatic colorectal cancer increasingly requires a multimodal approach and multiple treatment options based not on convenience, but in terms of personalization and efficacy. Therefore, it is important to optimize the pharmacokinetics of chemotherapeutic agents. Implantable port systems for colorectal cancer patients have been essential for oncological practice, and the importance of these systems will remain unchanged in the near future.

## Introduction

Over the past several decades, many management changes in oncology have occurred, especially in chemotherapy for colorectal cancer (CRC). From the early predominance of bolus injections of 5-fluorouracil (5-FU), chemotherapy has made considerable progress for CRC, including the biochemical modulation of the 5-FU effect by leucovorin, the development of infused 5-FU/leucovorin regimens and the introduction of irinotecan and oxaliplatin. Furthermore, the development of active cytotoxic chemotherapy regimens that incorporate biological targeted agents for metastatic CRC has significantly improved the survival periods of patients with metastatic CRC to more than 2 years [1–3]. Therefore, the main aims of modern chemotherapy for metastatic CRC have become the prolongation of survival and a better quality of life. In addition, modern chemotherapy using various combination regimens has become widely accepted as implantable port systems and disposable infusion pumps have been developed [4].

Easy-to-handle port systems are currently an important part of clinical practice in oncology. The history of chemotherapy using implantable port systems has three streams: (1) locoregional cancer therapy, (2) drug delivery and (3) implantable devices (Table 1). In the 1950s, there was increasing interest in the perfusion and infusion techniques for the application of chemotherapeutic agents, such as nitrogen mustard, in the locoregional treatment of metastatic cancer. Among them, treatment of liver metastasis has interested oncologists for many years, because it seems that, although the liver is a primary site of blood-borne metastases, it is often the only site of metastatic disease, especially in patients with CRC. Implantable devices have been further developed during the last few decades, and this has enabled patients with CRC with liver metastases to be treated effectively by locoregional therapy using hepatic arterial infusion (HAI); which became more common in the 1980s. With several lines of chemotherapy currently available for metastatic CRC, central venous catheters and implantable port systems are used worldwide. This article comprises a historical review of the progress made in chemotherapy for metastatic CRC from the viewpoint of the development of implantable port systems. It also discusses the current and future directions in chemotherapy for CRC using implantable port systems.

**Table 1:**
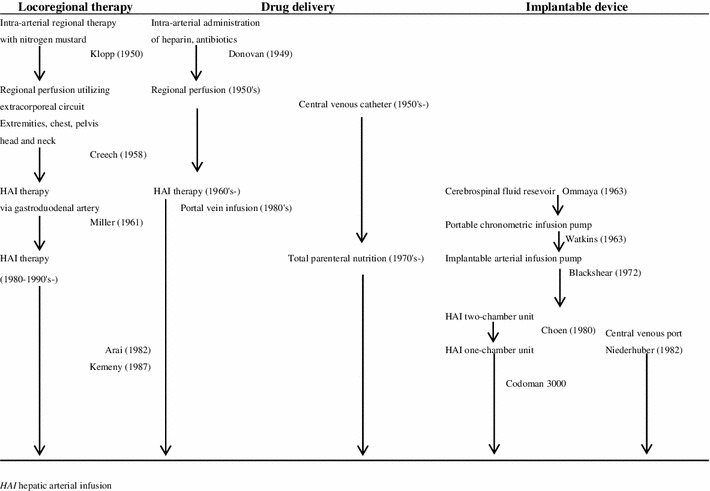
The history of implantable port systems

## History of locoregional cancer therapy

The first clinical application of cytotoxic chemotherapy for malignancies can be traced back to the 1940s, with the introduction of nitrogen mustard [5]. At that time, the limiting factor in the use of mustard compounds was their toxic effect on normal tissues. To avoid these systemic toxic effects, Klopp et al. [6] proposed the use of intra-arterial cancer chemotherapy with nitrogen mustard in 1950. A suitable technique was developed by utilizing a method devised for the intra-arterial administration of heparin [7], and polyethylene tubes were introduced into the external carotid artery. Next, the locoregional cancer therapy evolved into regional perfusion utilizing an extracorporeal circuit. In 1958, Creech et al. [8] employed the technique in 24 patients with various malignant neoplasms, and concluded that the administration of chemotherapeutic agents by perfusion is useful for localized tumors and for palliation of certain far-advanced lesions. As interest in locoregional infusion techniques increased, treatment of liver metastasis began to interest oncologists in the 1960s, because it seemed that although the liver is a primary site of blood-borne metastases, it is often the only site of metastatic disease, especially in patients with CRC.

**Fig. 1:**
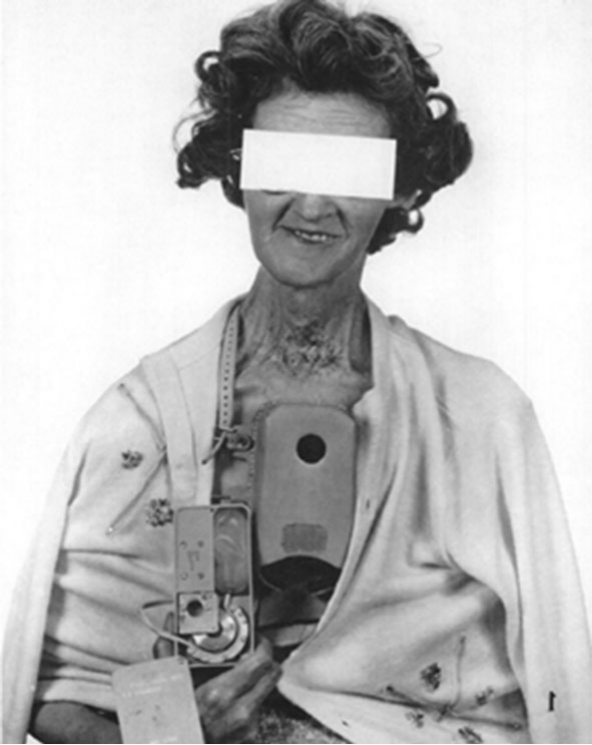
A chronometric ambulatory infusion apparatus. A disposable plastic bag serves as a reservoir for a concentrated solution of drug. Reprinted with permission from the American Association for Cancer Research [13]

While work with hepatic resection was proceeding, other groups were beginning to look carefully at the regional infusion of chemotherapy for liver metastases. However, hepatic arterial catheterization sometimes presented difficulties because of the extent of the disease. In 1961, a simple method of catheterization of the hepatic artery through the gastroepiploic and gastroduodenal arteries was first described by Miller et al. [9], who were the pioneers of the modern HAI technique. Since Watkins [10] developed the portable chronometric infusion pump to concentrate the administration of large doses of drugs to the liver in 1963, extensive clinical experience with regional infusion chemotherapy of hepatic tumors has been reported [11, 12]. Figure 1 shows a miniature portable chronometric infusion pump (Watkins USCI Chronofusor; United States Catheter & Instrument Corp., Glens Falls, New York) developed by Watkins et al. [10, 13]. Most experiences have involved percutaneously placed hepatic artery catheters connected to extracorporeal pumps. However, these systems have been associated with mechanical and infectious complications [14]. In the 1970s, a reliable, totally implantable device was developed for long-term intravascular drug infusion [14, 15]. The initial application of the pump was for long-term heparin infusions in patients with refractory thromboembolic disease [16]. With regard to cancer therapy, regional infusion of chemotherapy for liver metastases was expected to be oncologically useful. Thus, HAI chemotherapy for liver metastases from CRC became widely adopted in the 1980s.

## HAI chemotherapy for CRC

### Rationale for HAI

The rationale for hepatic arterial therapy is based on the fact that once liver metastases grow beyond 3 mm, they are fed predominantly by the hepatic artery, whereas normal hepatocytes are fed predominantly by the portal vein [17]. The liver may be the initial stop for metastatic spread through the portal vein, and early postoperative regional administration of chemotherapeutic agents into the portal vein might be particularly beneficial, destroying suspected tumor cells in the liver before established tumor growth can take place. However, from 1980 until the 1990s, several randomized trials failed to show significant differences in the survival benefit between portal vein infusion and systemic chemotherapy after curative surgery for advanced CRC [18–20].

### Clinical evidence of HAI chemotherapy for CRC

On the other hand, HAI for liver metastases from CRC was widely used based on several lines of evidence during the same period. HAI therapy is associated with not only anti-tumor effects, but also hepatic toxicity, and extensive studies have been performed to identify the optimal agents. Agents that are taken up by the liver and have high first-pass extraction, as well as high total-body clearance, are the most useful agents. The most active drug was shown to be 5-fluoro-2-deoxyuridine (FUDR), which has a 60–90 % first-pass liver extraction rate and an estimated 100- to 400-fold increase in hepatic exposure when used for HAI [21]. Several randomized trials from the 1980s to 2000 were performed to compare HAI using FUDR with systemic chemotherapy for unresectable liver metastases from CRC. The results suggested that HAI using FUDR was well tolerated and associated with a significantly better response rate compared with systemic chemotherapy using 5-FU in the treatment of metastases from CRC, although the survival benefit was limited. A meta-analysis combining the results of seven trials also supported the use of HAI using FUDR in the treatment of unresectable liver metastases from CRC [22]. A significantly higher response rate of 41 % was obtained with HAI using FUDR compared with a 14 % response rate with systemic 5-FU. In these previous reports, the median survival was significantly increased to 16 months with HAI versus 13 months with systemic treatment.

### Multimodal therapy using HAI for unresectable liver metastases

The hepatic resection criteria have been expanded with advances in surgical techniques; however, only 15–20 % of patients have disease that is suitable for resection at the time of presentation [23]. However, a proportion of tumors may become resectable after they respond to preoperative chemotherapy. Patients with potentially resectable liver metastases are now treated with conversion therapy using modern systemic chemotherapy, often a triple-drug cytotoxic regimen with molecular targeted agents, to maximally downsize the disease and subsequently facilitate curative surgical resection of the metastases [24, 25]. The conversion strategy stems from HAI therapy for liver metastases from CRC. HAI is characteristic in that high response rates can be achieved despite the fact that no modern active agents can increase the resection rates for initially unresectable liver metastases. Several studies have shown the efficacy of neoadjuvant HAI chemotherapy in patients with initially unresectable liver metastases from CRC [26]. High response rates of 16–90 % using HAI correlated with the complete hepatic resection rates. These results suggest that both locoregional drug delivery and optimization of the pharmacological effects are important for metastatic CRC therapy.

### Pharmacokinetics of HAI chemotherapy

The pharmacokinetics of 5-FU are influenced by the dose and schedule of administration. Short-term, high-concentration exposures are thought to favor RNA-directed 5-FU toxicity, whereas DNA-directed effects are felt to be more prominent with longer exposures to lower drug concentrations [27–29]. These pathways are not mutually exclusive, and more than one mechanism of action may contribute to cytotoxicity. This may support the recent standard intravenous 5-FU regimens comprising bolus plus infusional 5-FU in combination with leucovorin, derived from the DeGramont regimen [30]. Kusunoki et al. [31] reported another promising approach using oral fluoropyrimidines as pharmacokinetic modulating chemotherapy, the pharmacokinetic concept of which resembled that of the DeGramont regimen.

The concept of pharmacokinetic modulation is that the benefit of a continuous 5-FU infusion could be potentiated by low-dose oral uracil/tegafur (UFT), and the wide variation of 5-FU concentrations in each patient requires an implantable port system to achieve optimal concentrations. This pharmacokinetic modulating chemotherapy regimen has been proven to be highly effective for treating CRC [31, 32]. Its efficacy is based on the fact that the pharmacokinetic modulation targets at least two different phases of the cell cycle, depending on the dose of 5-FU [33]. HAI efficacy and other problems in patients with CRC with liver metastases have also been reported from a pharmacokinetic viewpoint [34]. In one study, HAI via an implantable port system comprised perfusion 5-FU for two consecutive days per week at 600 mg/m^2^/day and oral administration of UFT at 400 mg/day. HAI using pharmacokinetic modulating chemotherapy significantly decreased the incidence of hepatic recurrence after curative resection, but not the incidence of lung recurrence [34]. The authors stated that the maximum plasma concentration of 5-FU in the HAI group reached 144 ng/mL, which was lower than the 400 ng/mL reached in the systemic chemotherapy group, and discussed how the pharmacokinetics might lead to an oncological limitation of locoregional therapy. In other words, HAI chemotherapy might have an important role as an oncological strategy when combined with modern systemic chemotherapy or when used as part of multimodal therapy.

## Development of port devices

### Implantable port devices for HAI

**Fig. 2:**
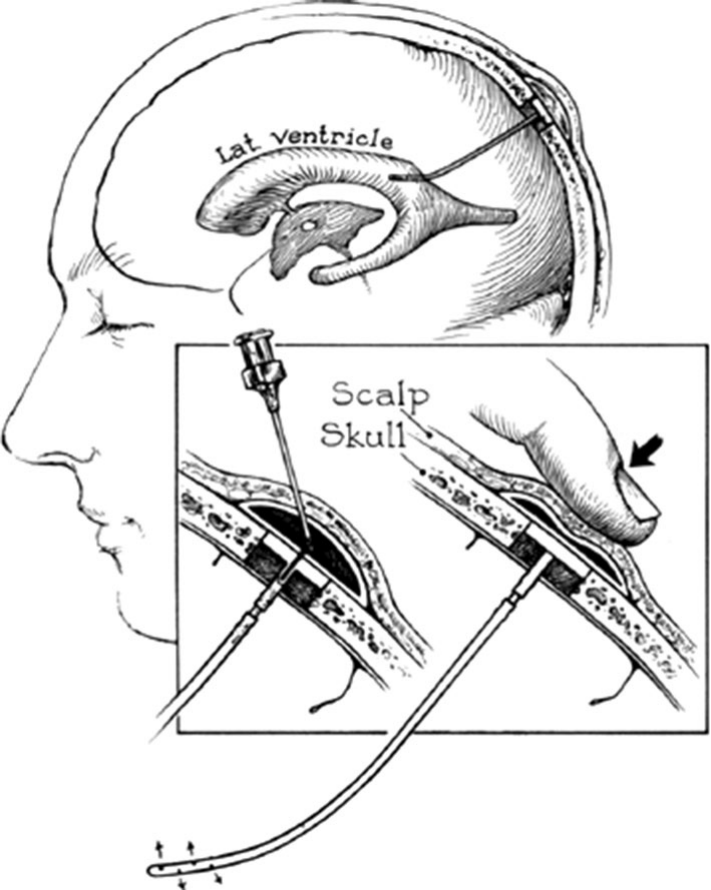
A diagram of a cerebrospinal fluid reservoir connected to the lateral ventricle, showing the method of use. Reprinted with permission from the Massachusetts Medical Society [36]

**Fig. 3:**
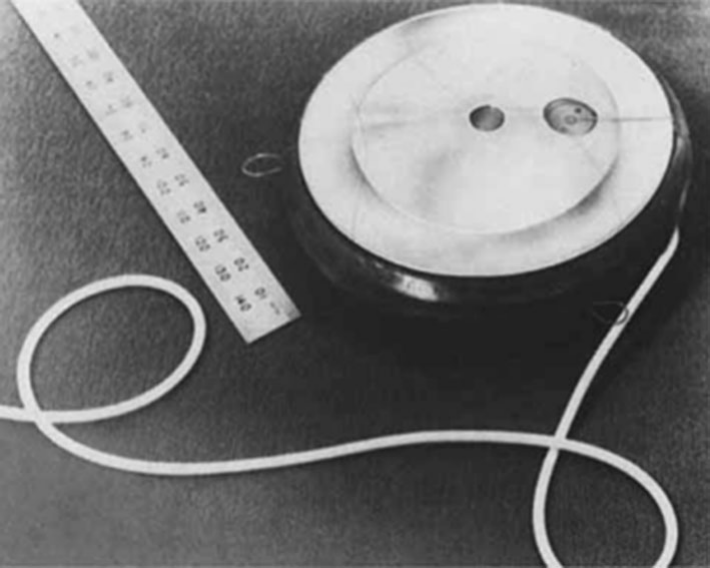
An implantable infusion pump. Dimensions: 2.82 cm height × 8.43 cm diameter. The empty weight was 184 g. This material is reproduced with the permission of the American Cancer Society and John Wiley & Sons, Inc [41]

The implantable port device was originally described in 1963 by Ommaya [35, 36] as a cerebrospinal fluid reservoir and manual pump (Fig. 2). The device was introduced to facilitate repeated injections of drugs into the cerebrospinal fluid of patients with fungal meningitides [37]. The device subsequently proved to be of considerable mechanical value in the treatment of malignant neoplasms of the nervous system by allowing the perfusion and instillation of cytotoxic agents [38, 39]. An implantable pump system that allows for continuous infusion of drugs, including heparin, was developed in 1972 [40]. The pump was a titanium disc about the size and shape of a small ice hockey puck (Fig. 3). The hollow titanium disc was divided into two chambers by a flexible titanium bellows. The first use of this device to deliver chemotherapeutic agents to hepatic tumors was reported in 1980 [41]. It proved very reliable, without mechanical complications, and was rarely subject to the problems seen with percutaneously placed catheters that are left in place for prolonged infusion. After the implantable port device went into production in the 1970s, HAI was more commonly performed.

**Fig. 4:**
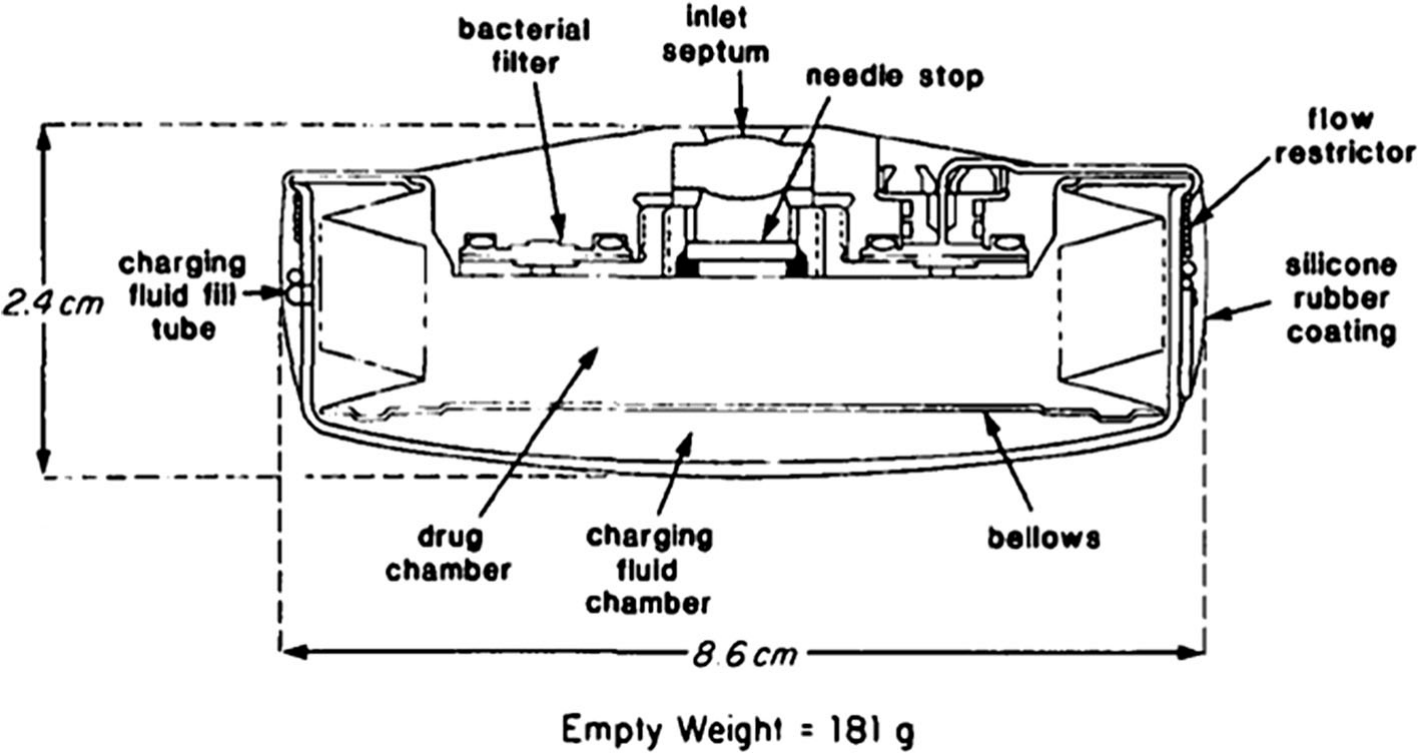
A cross-sectional diagram of the Infusaid^®^ pump. Reprinted with permission from the American Diabetes Association [43]

**Fig. 5:**
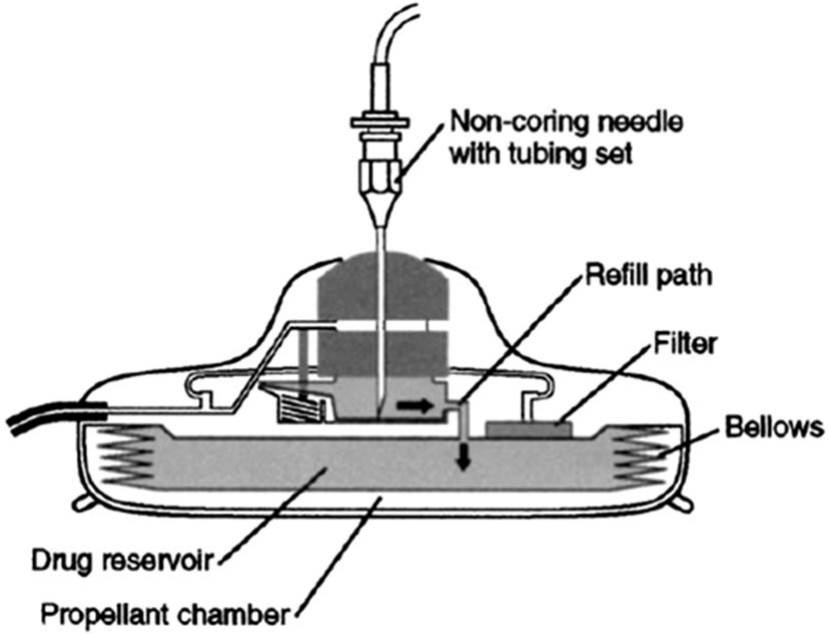
A schematic diagram of an implantable pump. Reprinted with permission from Elsevier Ltd. [44]

The development of the non-battery-powered mechanism for a totally implantable HAI pump allowed for the use of HAI as a long-term therapy. In 1980, Cohen et al. [42] reported transbrachial hepatic arterial chemotherapy using a modern implantable infusion pump. The Infusaid^®^ implantable pump (Metal Bellows Corporation, Sharon, Massachusetts) was a hockey-puck-sized (9 cm in diameter, 2 cm thick) titanium shell encasing welded titanium. The implantable pump had a 50-cm^3^ reservoir and a side port by which one could directly inject drugs into the catheter. The basic design of the pump was a two-chambered unit made of titanium [43] (Fig. 4). One chamber was a drug fluid chamber that could be accessed from the outside of the pump. The other chamber was charging fluid chamber that was filled with Freon. The mechanism driving the pump involved mechanical energy that was supplied during each refill of the pump. The fluid to fill the pump would be placed into the drug chamber by means of a percutaneously placed needle. This would fill up the drug chamber and push out the bellows that compressed the charging chamber. The compressed Freon would also expand with body heat, then exert its energy by pushing up on the diaphragm in the device, which would slowly push fluid out through the catheter of the pump. After the Infusaid^®^ implantable pump was developed, other pump designs with different mechanisms appeared to further miniaturize these devices, some of which could only be accessed from the central needle inlet [44]. The Codman Constant Flow Infusion Pump was designed with a raised septum at the center of the device that can be easily palpated through the skin, making the refill septum more accessible (Fig. 5). This also allowed for much easier detection should a pump invert within the pocket. The safety of use and the efficacy of treatment for regional chemotherapy and pain control were reported previously [45–48].

HAI treatment was initially performed by means of a catheter that was surgically placed in the hepatic artery after isolation of the gastroduodenal artery, and the catheter was then connected to the port placed in a subcutaneous pocket [49, 50]. To access the artery more easily, Arai et al. [51] reported a radiological method with which to place an implantable catheter by surgical cut-down of the small branch of the subclavian artery in 1982. In 1990, Kumada et al. [52] also reported a technique created by modifying Arai’s method so that arterial access could be directly obtained under sonographic guidance. These minimally invasive ways of implanting an infusion catheter for chemotherapy contributed to the widespread use of HAI treatment for liver metastases from CRC.

### Central venous port systems

Historically, implantable port systems were developed without distinction between hepatic arterial or central venous access. However, there was a difference in the original aim of drug delivery systems between HAI and central venous access. Whereas HAI via implantable port systems was developed for long-term oncological treatment, central venous port systems were developed for several reasons, including repeated administration of chemotherapeutic agents, parental nutrition, transfusions, infusions, injections and/or blood sample collection. Consequently, central venous port systems play a key role in modern oncology, especially in chemotherapy for patients with metastatic CRC. Frequent puncturing of peripheral veins and the local effects of chemotherapeutic drugs cause damage, thrombosis and sclerosis of vascular walls, and the use of port systems for permanent central venous access allows patient to continue long-term treatment.

The first central venous catheters developed were percutaneous, non-tunneled catheters, which came into use in the 1950s [53]. The first long-term central venous catheter was used for parenteral nutrition in 1973 [54]. With regard to oncological use, the Hickman [55] catheter, a long-term venous access device, was used for chemotherapy for the first time in 1979. Open, tunneled central venous catheter systems, such as the Hickman catheter, are associated with a higher infection rate because the end of the catheter remains outside the body. Implantable port systems have advantages in that the puncturing needle can be removed after each injection, and the skin covering the port serves as a natural protection against infection. The currently used central venous port system was introduced to clinical use in 1982 by Niederhuber et al. [56]. The device, an injection port (Infuse-A-Port; Infusaid Corp., Sharon, Massachusetts), comprises a 3.5 × 1.5 cm conical chamber with a self-sealing silicon rubber septum connected to a Silastic catheter. This totally implanted venous and arterial access system was initially tested in 30 patients with cancer. A variety of anticancer agents was administered without difficulty, and patient acceptance was excellent.

The Infuse-A-Port had advantages in terms of its size and cost compared with the above-mentioned Infusaid^®^ implantable pump with its complex mechanism. In the early 1980s, both the Infuse-A-Port and Infusaid^®^ implantable pumps were introduced in Japan by Miura et al. [57]. Over the last few decades, central venous port systems have been implanted during minimally invasive procedures by surgeons or interventional radiologists. Implantable central venous port systems therefore facilitate safe and easy blood access in oncology today, although several relevant long-term complications exist, as described below.

### Complications associated with central venous ports

There are several important complications associated with central venous ports [58, 59]. Port complications can be subdivided into procedural complications, catheter-related complications and vascular complications. Short-term complications include accidental arterial puncture, hematoma, air embolism, pneumothorax or vessel perforation [60], but these complications are rare in modern oncology. Surgical complications arise in <2 % of cases in experienced hands [61]. Clinical oncologists are currently most often concerned with major long-term complications associated with the use of catheters in chemotherapy. A retrospective study by Yildizeli et al. [62] showed that among 225 implantable port systems, the long-term complications included infection (2.2 %), thrombosis (1.3 %), extravasation (1.3 %) and catheter fracture (2.2 %). The major long-term problems of catheter use in patients with cancer are catheter-related infection and thromboembolic complications. Both complications may lead to significant morbidity and impairment of the patient’s quality of life.

The catheter-related infection rates in recent studies ranged from 0.8 to 7.5 % [63, 64]. The incidence of catheter-associated thrombosis in patients with cancer varies considerably between studies and patient or cancer type, with the incidence of catheter-associated thrombosis among several studies varying widely from 12 to 64 % [65–69]. Several researchers evaluated the benefit of anticoagulant prophylaxis with either low molecular weight heparin or warfarin in patients with cancer using central venous devices; however, routine anticoagulation cannot be recommended [69–73].

## Importance of pharmacokinetics in the chemotherapy for CRC

Even in modern chemotherapy using several active agents, 5-FU plays a central role in the treatment of CRC, and many attempts have been made to improve its efficacy and potentiate its action over the last 50 years. In particular, recent reports suggested that the pharmacokinetics of 5-FU remain an important factor that affects the prognosis of patients with CRC, even after the introduction of modern chemotherapy for CRC [74–77]. An implantable port system is indispensable in both HAI and central venous port systems to optimize the pharmacokinetics in multiagent chemotherapy. This is important, because the treatment of advanced CRC increasingly requires a multimodal approach. A recent trend highlights the possibility of using active first-line chemotherapy to affect the downstaging of metastases and so enable curative surgery for initially unresectable disease, especially liver metastases from CRC [24, 25, 78]. From a pharmacological viewpoint, both dose- and time-dependent administration of chemotherapeutic agents is possible via implantable port systems, thus resulting in high response rates in many situations.

On the other hand, recent attention has been given to port-free chemotherapy using oral fluoropyrimidines, such as capecitabine, because of their convenience. Capecitabine is a unique fluoropyrimidine carbamate that is selectively converted to 5-FU in tumors through a cascade involving three enzymes [79]. Using a rational design and taking advantage of the unique tissue localization patterns of these key enzymes, capecitabine was developed to be selectively activated within tumor tissues. Indeed, CapeOx with bevacizumab as port-free chemotherapy showed high response rates ranging from 50 to 78 % and a high conversion rate of 40 % for initially unresectable hepatic metastases [80, 81]. However, recent evidence shows that there are few choices regarding port-free chemotherapy, especially first-line chemotherapy, for advanced or metastatic CRC. Besides CapeOx with bevacizumab, the other recommendations for first-line chemotherapy for advanced or metastatic CRC in the NCCN guidelines include FOLFOX ± bevacizumab, FOLFIRI ± bevacizumab, FOLFIRI ± ant-EGFR antibody, 5-FU + leucovorin + bevacizumab and FOLFOXIRI; all require central venous port systems [82].

## Conclusion

Modern chemotherapy using various combinations of regimens for advanced CRC became widely accepted with the development of the implantable port system. Historically, chemotherapy using implantable port systems was begun for the purpose of locoregional cancer therapy to obtain further oncological benefits. This concept resembles that of modern chemotherapy, which aims to optimize the pharmacokinetics of chemotherapeutic agents. The treatment of advanced CRC increasingly requires a multimodal approach and multiple treatment options, which are based not only on convenience, but especially on personalization and efficacy. Implantable port systems for CRC have been essential for oncological practice, and the oncologic importance of these systems will remain unchanged in the near future.
